# The mitochondrial genome of *Conosia irrorata* (Diptera: Limoniidae)

**DOI:** 10.1080/23802359.2019.1631130

**Published:** 2019-07-12

**Authors:** Bing Zhang, Shang Gao, Ding Yang

**Affiliations:** Department of Entomology, College of Plant Protection, China Agricultural University, Beijing, China

**Keywords:** Mitochondrial genome, Limnophilinae, phylogenetics

## Abstract

The crane fly *Conosia irrorata* belongs to the subfamily Limnophilinae of Limoniidae. The mitogenome (GenBank accession number: MK864103) of *C. irrorata* was sequenced, the new representative of the mitogenome of the subfamily. The nearly complete mitogenome is 14,634 bp totally, consisting of 13 protein-coding genes, two rRNAs, and 22 transfer RNAs. All genes have the similar locations and strands with that of other published species of Limoniidae. The nucleotide composition biases toward A and T, which together made up 77.5％ of the entirety. Bayesian inference analysis strongly supported the monophyly of Tipuloidea. It suggested that the monophyletic Tipulidae was assigned to the sister to the monophyletic Cylindrotomidae. The Limoniidae is the sister group to the clade of Tipulidae + Cylindrotomidae, and Pediciidae was assigned to the sister to the group that contain Limoniidae + (Tipulidae + Cylindrotomidae).

## Introduction

The subfamily Limnophilinae belongs to Limoniidae, with over 2700 known (sub)species worldwide (Oosterbroek 2018).

The specimens of *C. irrorata* used for this study were collected in Longchuan County (24°10'N, 97°49'E) of Yunnan Province by Bing Zhang in 2018, and then identified by Bing Zhang. Specimens are deposited in the Entomological Museum of China Agricultural University (CAU). The total genomic DNA was extracted from the whole body (except head) of the specimen using the QIAamp DNA Blood Mini Kit (Qiagen, Germany) and stored at −20 °C until needed. The mitogenome was amplified and sequenced as described in our previous study (Wang, Li, et al. 2016). The nearly complete mitogenome (GenBank accession number: MK864103) of *C. irrorata* is 14,634 bp. It encoded 13 PCGs, 22 tRNA genes, and two rRNA genes and the control region could not be sequenced entirely in this study, and were similar with related reports before (Wang, Ding, et al. 2016; Wang, Wang, et al. 2016; Li et al. 2017; Zhou et al. 2017; Qilemoge et al. 2018). All genes have the similar locations and strands with that of other published Limoniidae species. The nucleotide composition of the mitogenome was biased toward A and T, with 77.5% of A + T content (A = 39.8%, T = 37.7%, C = 13.6%, and G = 8.9%). The A + T content of PCGs, tRNAs, and rRNAs is 76.7%, 79.3%, and 80.8%, respectively. The total length of all 13 PCGs of *C. irrorata* is 11,238 bp. Two PCGs (*NAD2* and *NAD6*) initiated with ATT codons, and seven PCGs (*COII*, *COIII*, *ATP6*, *ATP8, NAD4, NAD4L,* and *CYTB*) initiated with ATG codons, *NAD1* initiated with ATA as a start codon, *CO1* and *NAD5* initiated with TTG and GTG as a start codon, respectively. Ten PCGs used the typical termination codons TAA, one PCG (*CYTB*) used TAG, and two PCGs (*NAD4* and *NAD5*) used TA and T in *C. irrorate,* respectively.

**Figure 1.**
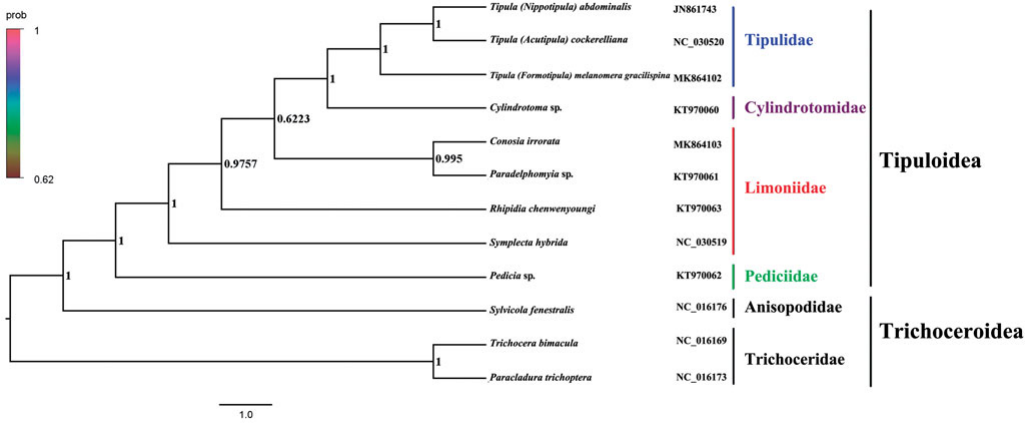
Bayesian phylogenetic tree of 12 Diptera species. The posterior probabilities are labeled at each node. Genbank accession numbers of all sequence used in the phylogenetic tree have been included in the Figure 1 and corresponding to the names of all species.

Phylogenetic analysis was performed based on the nucleotide sequences of 13 PCGs from 12 Diptera species. Bayesian (BI) analysis generated the phylogenetic tree topology based on the PCGs matrices (Figure 1). According to the phylogenetic result, it showed that the monophyletic Tipuloidea was assigned to be the sister group to the clade of Trichoceroidea with Trichoceridae and Anisopodidae in this text. The monophyletic Tipulidae was the sister group to the monophyletic Cylindrotomidae. For the phylogeny of Tipuloidea, the clade consisting of Tipulidae + Cylindrotomidae is the sister group to the clade of Limoniidae and Pediciidae was assigned to the sister to the group that contains Limoniidae + (Tipulidae + Cylindrotomidae). The phylogenetic relationship inferred from the phylogenetic result in this text is very clear: (Trichoceridae + Anisopodidae) + (Pediciidae + (Limoniidae + (Tipulidae + Cylindrotomidae))). The relationship of the sister group within Tipuloidea was also supported by the previous study (Petersen et al. 2010). The mitogenome of *C. irrorata* could provide the important information for the further studies of Tipuloidea phylogeny.
